# Small subunit isoform diversity underlies structural heterogeneity in native plant Rubisco

**DOI:** 10.1073/pnas.2519949123

**Published:** 2026-04-15

**Authors:** Thomas Reynolds, Zhemin Zhang, Dušan Živković, Steven Kelly, Jani R. Bolla

**Affiliations:** ^a^Department of Biology, Univeristy of Oxford, Oxford OX1 3RB, United Kingdom; ^b^Department of Pharmacology, Case Western Reserve University, Cleveland, OH 44106; ^c^Department of Biochemistry, University of Oxford, Oxford, OX1 3QU, United Kingdom

**Keywords:** Rubisco, cryo-EM, native mass spectrometry, *Arabidopsis thaliana*, structural heterogeneity

## Abstract

Ribulose-1,5-bisphosphate carboxylase/oxygenase (Rubisco), the most abundant protein on Earth, catalyzes the fixation of CO_2_ in photosynthesis. In terrestrial plants, Rubisco assembles as a hexadecameric complex (L_8_S_8_), comprising eight large subunits (LSu) and eight small subunits (SSu). While LSu is encoded by a single chloroplastic gene, a nuclear multigene family results in diverse SSu protein isoforms, but structural evidence is currently lacking for Rubisco holoenzyme SSu heterogeneity. In this study, utilizing native Rubisco purified from *Arabidopsis thaliana*, we employed high-resolution mass spectrometry and cryo–electron microscopy to demonstrate that multiple SSu isoforms can co-assemble within individual Rubisco complexes. We unambiguously identify the composition of these mixed-isoform complexes and elucidate isoform-specific structural interactions at near-atomic resolution. The structural heterogeneity in plant Rubisco established here will underpin future research to establish the impact of SSu diversity on kinetic and functional plasticity of Rubisco activity under diverse environmental conditions.

## Significance

Ribulose-1,5-bisphosphate carboxylase/oxygenase (Rubisco), the key enzyme in photosynthetic CO_2_ fixation, has long challenged efforts to understand its assembly, regulation, and potential for enhancement. Here, we show that multiple small subunit (SSu) isoforms can coexist within individual Rubisco complexes in *Arabidopsis thaliana*. Combining native protein extraction, high-resolution mass spectrometry, and cryo–electron microscopy, we define these mixed-isoform assemblies and reveal how different SSu variants shape local structural environments. Previous studies have demonstrated that plants maintain diverse SSu gene families and suggest that isoform composition might fine-tune Rubisco activity in response to developmental and environmental changes. Our results provide fundamental insights into Rubisco composition at the holoenzyme level and a structural framework for interpreting differences in Rubisco activity arising from varying isoform compositions.

Photosynthesis underpins almost all life on Earth, converting carbon dioxide (CO_2_) and water into sugars and oxygen using sunlight. Ribulose-1,5-bisphosphate carboxylase/oxygenase (Rubisco) catalyzes the first step of CO_2_ fixation (1). Rubisco is both essential and inefficient; its slow turnover and competing oxygenase reaction limit photosynthetic output, but its abundance compensates for these limitations (2, 3). In land plants, Rubisco is a hexadecameric (L_8_S_8_) complex composed of eight chloroplast-encoded large subunits (LSu) and eight nucleus-encoded small subunits (SSu). Nucleus-encoded subunits are translated in the cytosol as preproteins with an N-terminal chloroplast-targeting sequence, called a transit peptide, which is cleaved and degraded after importing preproteins into the chloroplast through translocons of the outer and inner chloroplast membrane (TOC and TIC, respectively) (4, 5). Once folded in the stroma, the SSu is complexed with folded LSu, assisted by chaperones, to form the functional holoenzyme, L_8_S_8_ (*SI Appendix*, Fig. S1*A*). The large subunits form the catalytic core, whereas the small subunits cap its ends and are required for correct folding, assembly, and function. LSu is strikingly conserved among higher plants, but the small subunits are not. Most species express multiple SSu isoforms, ranging from a few to more than twenty (6). Differential expression of these isoforms across tissues and environmental conditions has been reported in *Arabidopsis*, tomato, tobacco, rice, and maize (6), suggesting a regulatory complexity whose mechanistic basis remains poorly understood.

Although SSu does not directly participate in catalysis, it plays an essential role in Rubisco biogenesis, structural stability, and catalytic efficiency. SSu contributes to enzyme assembly, enhances CO_2_ binding efficiency, and may serve as a reservoir for CO_2_ (6–8). In several cases, replacing the native SSu with a heterologous isoform has been sufficient to alter carboxylation rates and specificity (8–12). However, the structural determinants of this functional variability remain obscure, largely due to an incomplete understanding of how different SSu isoforms interact with LSu and influence enzyme activity. A fundamental question also remains unresolved: Do individual L_8_S_8_ complexes in planta contain a single small-subunit isoform, or can multiple isoforms coexist within the same particle?

Over the past decades, increasing efforts have focused on Rubisco biogenesis, structural stability, and engineering, motivated by the urgent need to improve photosynthetic efficiency in crops under rising global demands and climate stress (6, 7, 13). To guide these investigations, several Rubisco structures have been solved at near-atomic resolution, revealing molecular details of protein–protein interactions within Rubisco. However, nearly all available models contain only a single SSu isoform, likely due to crystal packing constraints. Only two studies to date report any heterogeneity within the L_8_S_8_ complex (14, 15), but no heterogeneous structures have been deposited in the protein databank (PDB), reinforcing the prevailing assumption that Rubisco incorporates only one SSu isoform per assembly, despite clear evidence that multiple isoforms are expressed simultaneously in most plants and their relative abundance shifts with changing environmental and developmental context (6, 16). Under such circumstances, it is not obvious why every Rubisco complex should be compositionally identical (17, 18). Until very recently, the available methodological toolkit was not well-suited to answering this question decisively.

Clarifying the true composition of plant Rubisco is important for both fundamental biology and practical applications. Most engineering strategies, whether importing faster algal Rubiscos into crops (several crop species also express various SSu isoforms, see *SI Appendix*, Fig. S2 for a few examples) or redesigning plant small subunits to improve CO_2_ specificity, focus on introducing a single “superior” SSu into plants. However, it is not yet known if native Rubisco typically incorporates a mixture of SSu isoforms with distinct structural roles, and the potential structure–function consequences of mixed populations have not yet been considered. For example, it is not known whether natively produced heterogeneous Rubisco would be beneficial, detrimental, or neutral for Rubisco assembly, stability, function, or regulation. To explore the extent and structural ramifications of heterogeneous Rubisco, we combined high-resolution cryo–electron microscopy (cryo-EM) with mass spectrometry (MS) to examine Rubisco purified directly from the model plant, *Arabidopsis thaliana*. MS unambiguously identifies the presence of multiple SSu isoforms, while cryo-EM resolves their spatial arrangement and isoform-specific interactions within the holoenzyme. These findings challenge the long-standing model of isoform exclusivity and establish that mixed assemblies occur *in planta*.

## Results

### Isolation of Intact Rubisco from Plant Leaves.

**Fig. 1.**
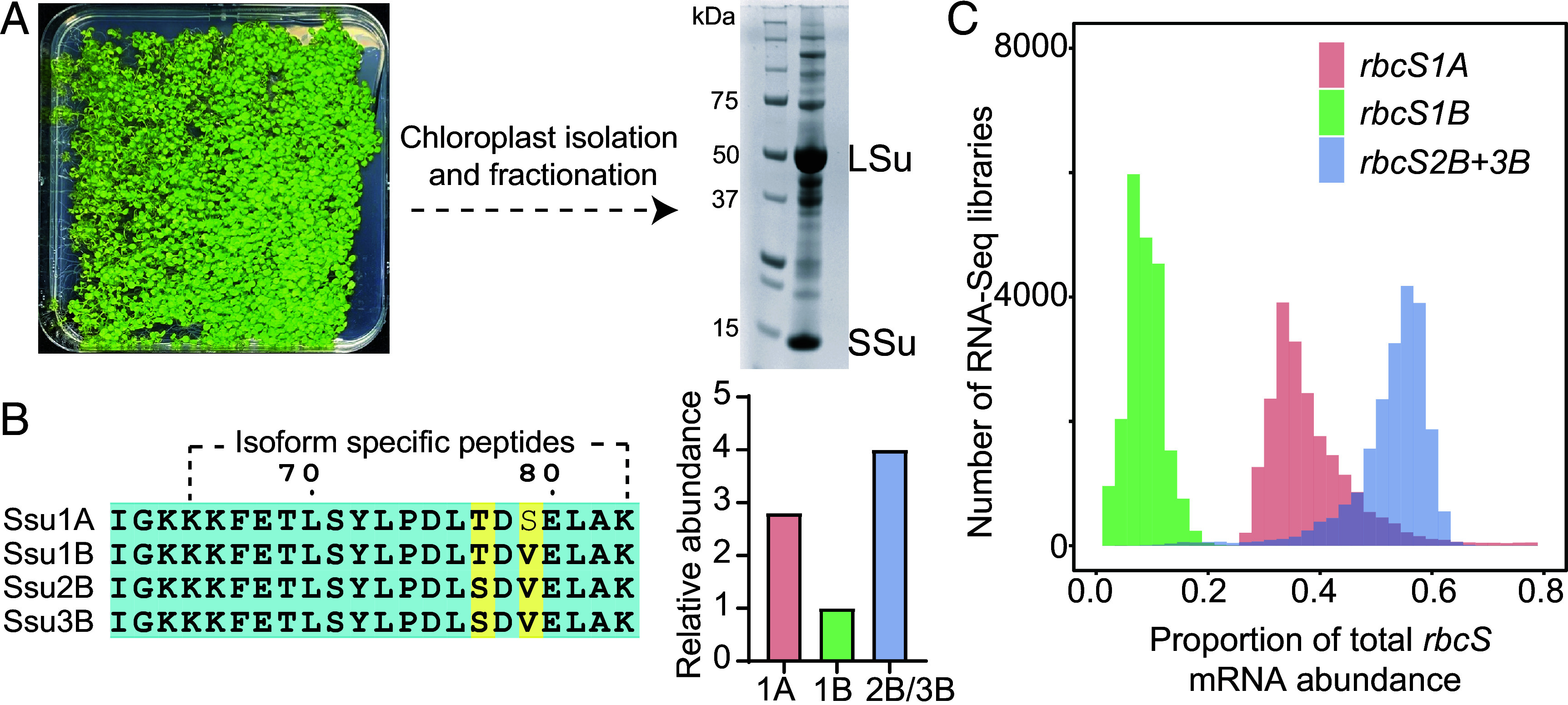
Native Rubisco contains multiple SSu isoforms. (*A*) Leaves from 14-d-old *A. thaliana* plants were used for chloroplast isolation, followed by fractionation to obtain the soluble stromal fraction. SDS–PAGE (*Right*) shows that Rubisco is abundant in this stromal fraction. Notably, this workflow was designed to enrich the chloroplast stroma rather than to purify Rubisco to homogeneity, thereby reducing the risk of losing protein subpopulations during more stringent purification. (*B*) Regions of the SSu sequence containing isoform-specific peptides identified by proteomics, and their relative quantification (bar graph, *Right*) is consistent with the *rbcS* expression profile across 21,154 publicly available RNA-seq libraries (*C*).

To investigate the native assembly of Rubisco and assess whether distinct SSu isoforms can coexist within a single holoenzyme, we purified Rubisco from *A. thaliana.* Chloroplasts were isolated from 14-d-old plants and lysed using a hypotonic solution. The stroma of lysed chloroplasts was obtained using previously established fractionation methods (19). The stromal soluble fraction was further concentrated using a 100 kDa molecular weight concentrator to enrich large complexes. SDS–PAGE analysis showed that the preparation was dominated by Rubisco, with prominent bands at ~55 kDa and ~15 kDa corresponding to the LSu and SSu bands (Fig. 1*A* and *SI Appendix*, Fig. S3). To verify that the purified enzyme remained intact and correctly assembled, we performed mass photometry, a label-free single-molecule technique that enables stoichiometric estimation of protein complexes in solution (*SI Appendix*, Fig. S4). The resulting histogram revealed a major symmetric peak centered at approximately 541 kDa, consistent with the theoretical molecular mass of an L_8_S_8_ complex. These results indicate that the isolation of intact Rubisco from plant leaves preserved Rubisco’s native architecture.

### Isolated Rubisco Contains Multiple SSu Isoforms.

*A. thaliana* expresses four SSu isoforms (SSu1A, SSu1B, SSu2B, and SSu3B) (*SI Appendix*, Fig. S1*B*). The mature forms of SSu2B and SSu3B are identical (henceforth denoted SSu2B/3B in this paper). To identify which of these isoforms are present in our Rubisco preparation, we performed bottom–up proteomics. This approach allowed us to detect isoform-specific peptides of SSu (*SI Appendix*, Fig. S3), and their relative quantification is shown in Fig. 1*B*. When considered semiquantitatively, the relative intensities of isoform-specific peptides are broadly consistent with the *rbcS* expression patterns observed across 21,154 publicly available RNA-seq libraries (with SSu2B and SSu3B aggregated because they are indistinguishable at the protein level). After aggregating the nearly identical SSu2B and SSu3B transcripts, expression profiles revealed that SSu2B/3B dominate in most photosynthetic tissues, whereas SSu1A and SSu1B are more variably expressed across developmental stages and environmental conditions (Fig. 1*C*). The close concordance between transcript abundance and proteoform representation strongly suggests that the detected mixture of SSu isoforms reflects bona fide in planta diversity rather than adventitious association or contamination. Together, these findings indicate that *A. thaliana* Rubisco does not exist as a homogeneous population of identical L_8_S_8_ complexes. Instead, it comprises a heterogeneous ensemble of assemblies that incorporate various combinations of SSu isoforms, potentially expanding the enzyme’s functional versatility in planta.

### SSu1A and SSu2B/3B Are the Two Stably Binding Isoforms.

**Fig. 2.**
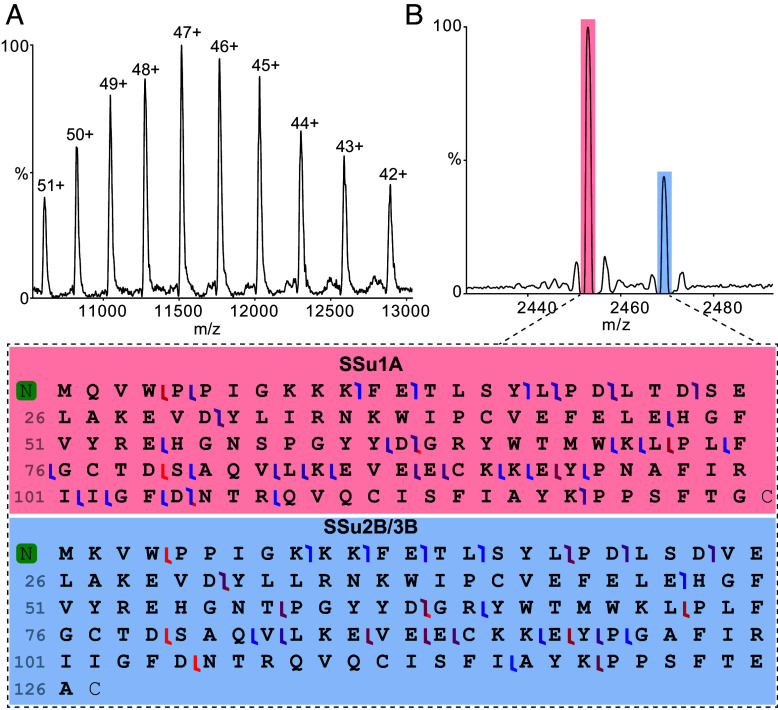
Top–down MS reveals the cooccurrence of two SSu isoforms in the native Rubisco population. (*A*) Native MS of Rubisco yields a charge-state envelope that deconvolutes to a measured mass of 541,220 ± 19 Da, consistent with the intact L_8_S_8_ holoenzyme, confirming that the complex remains assembled during analysis. Charge states are labeled for clarity. (*B*) Increasing collisional activation dissociates SSu subunits from the holoenzyme, yielding two SSu signals at ~2,453 and ~2,469 m/z. Top–down fragmentation (using HCD) of these SSu ions generates isoform-specific fragment ions that uniquely identify SSu1A and SSu2B/3B. Together, these data show that the native Rubisco population contains more than one SSu isoform.

To assess whether the above-identified SSu isoform ratios reflect stable incorporation into L_8_S_8_ complexes, rather than weak or transient association, we turned our attention to high-resolution native MS coupled with in-source dissociation and top-down tandem fragmentation (MS/MS). This approach allows detection of intact proteoforms, their posttranslational modifications, and sequence variations without denaturation or proteolytic digestion. We first analyzed the above purified stromal soluble fraction, which predominantly contained Rubisco, by native MS, and the resultant spectrum revealed a major charge-state series whose mass corresponded to the intact L_8_S_8_ complex (Fig. 2). Next, we applied in-source activation energy to release intact SSu monomers, namely m/z corresponding to 2,453 and 2,469, which were subsequently isolated and fragmented using higher-energy collisional dissociation (HCD). The resulting spectra contained a plethora of fragment ions that yielded unambiguous sequence coverage for SSu1A and SSu2B/3B proteoforms (Fig. 2 and *SI Appendix*, Fig. S5), with weaker signals from SSu1B. Of note, all “B” isoforms (1B, 2B, 3B) of SSu share the same molecular weight. The relative signal intensities of these species suggest a nonstoichiometric but reproducible representation of multiple isoforms within the same preparation. Interestingly, all detected SSu proteoforms displayed an N-terminal methylation, a conserved modification previously reported in spinach and tobacco Rubisco, that has been proposed to enhance holoenzyme stability (20).

### Spatial Distribution of SSu Isoforms around the L_8_ Core Is Disordered.

**Fig. 3.**
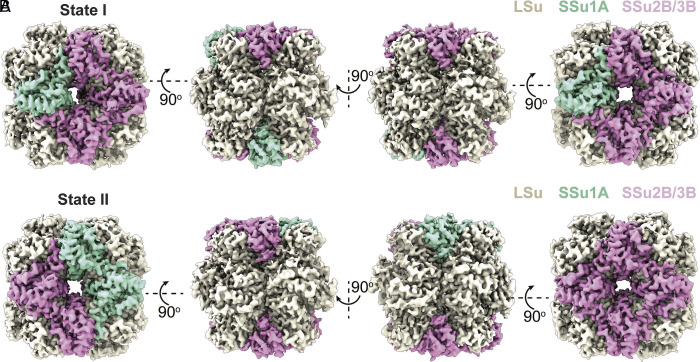
Cryo-EM analysis reveals two distinct heterogeneous architectures of Rubisco. Cryo-EM reconstructions of the native enzyme identify two structural states, State I (*A*) and State II (*B*), each represented as surface views from multiple orientations (*Top* view—*Left*, *Bottom* view—*Right*). LSu are shown in beige, SSu1A in green, and SSu2B/3B in magenta. The two states differ in both the composition and spatial arrangement of SSu1A and SSu2B/3B around the L_8_ core, demonstrating that native Rubisco exists as a mixture of distinct isoform-containing assemblies.

To directly visualize how different SSu isoforms are arranged within the holoenzyme, we subjected the same Rubisco preparations from above to cryo-EM analysis. Over 250,000 particle images were collected and processed using single-particle reconstruction workflows, yielding two major structural states (state I and II) (Fig. 3 and *SI Appendix*, Fig. S6 and Table S1), each characterized by a distinct composition and spatial distribution of SSu isoforms. In both states, the overall architecture resembles previously reported structures (16). Briefly, the large subunits form an L_8_ core made up of a tetramer of dimers, with four small subunits forming cap-like coverings at the top and four capping the bottom of this barrel-shaped LSu core. Focused refinement of the small-subunit regions, however, revealed subtle but reproducible density differences consistent with compositional heterogeneity. In State I, each end of the core is capped by one SSu1A and three SSu2B/3B subunits, while in State II, the upper cap contains two SSu1A and two SSu2B/3B subunits, and the lower cap consists exclusively of four SSu2B/3B (Fig. 3). These observations are fully consistent with the relative isoform abundances observed by MS, providing orthogonal validation of mixed-isoform assembly.

### Isoform-Specific Residues Influence Rubisco Core Stability.

**Fig. 4.**
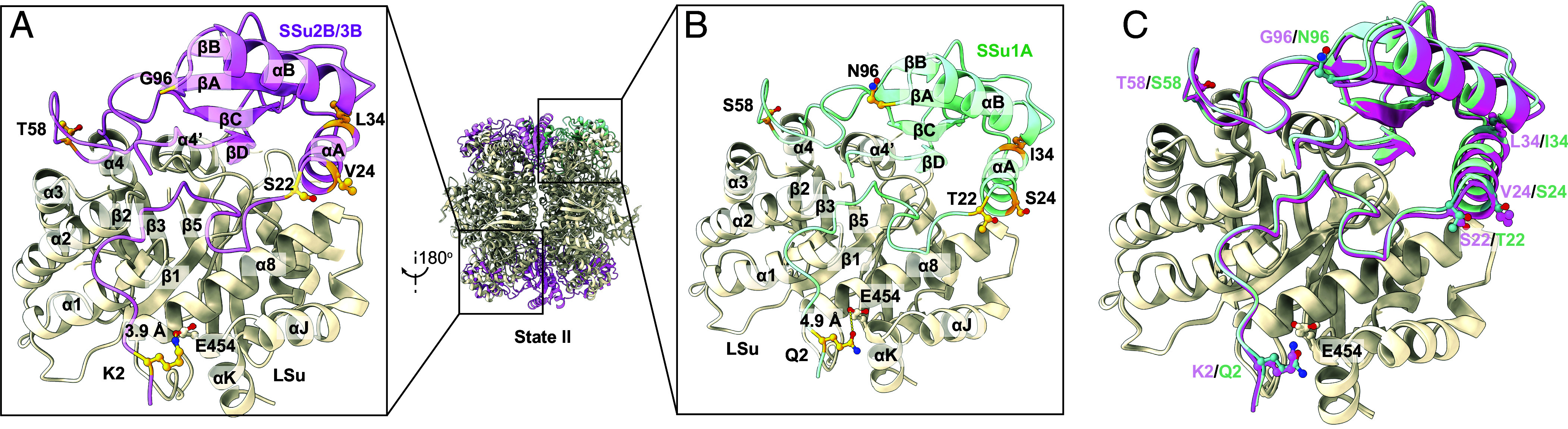
Isoform-specific interactions at the Rubisco LSu–SSu interface in State II. (*A*) Zoomed-in view of the interface between the LSu (beige) and SSu2B/3B (magenta), highlighting isoform-specific contacts at the N terminus and β-barrel of the SSu. Selected interacting residues are shown as sticks, with putative hydrogen bonds/salt bridges indicated by dashed lines and distances. (*B*) Corresponding view of the LSu–SSu1A interface (LSu in beige, SSu1A in teal), illustrating alternative side-chain identities and geometries at analogous positions that give rise to a distinct interaction network. (*C*) Overlay of the LSu–SSu2B/3B (magenta) and LSu–SSu1A (teal) complexes, showing that the LSu backbone is largely conserved while the different SSu isoforms adopt subtly shifted orientations and side-chain conformations at the interface. Rubisco secondary-structure elements are labeled according to the established convention (19).

SSu1A and SSu2B/3B differ in seven positions: Q2K, T22S, S24V, I34L, S58T, N96G, and G124E (*SI Appendix*, Fig. S1*B*). Our cryo-EM density maps enabled confident assignment of side-chain identities at six of the seven positions distinguishing SSu1A from the B-type isoforms (Q2K, T22S, S24V, I34L, S58T, N96G) (*SI Appendix*, Fig. S7). The substitutions cluster at two interfaces: near the C-terminal β-barrel of SSu and the α8–αK helices of LSu, which together shape the catalytic pocket (Fig. 4 and *SI Appendix*, Fig. S8). Notably, residue 2 in SSu (Q in SSu1A, K in SSu2B/3B) sits within 5.5 Å of LSu helix α8 and within 3.9 to 4.6 Å of E454 on helix αK. The lysine present in SSu2B/3B is therefore well positioned to form a stabilizing salt bridge with E454, which could, in turn, influence the conformational dynamics of loop 6 during CO_2_ fixation (21). Given the central role of loop 6 in carboxylation chemistry, even such minor alterations may modulate catalytic turnover or substrate affinity.

The conservative serine-to-threonine substitution at position 58 occurs on the βA–βB loop that projects into the solvent channel. Although the polarity is retained, the additional methyl group in threonine may reduce flexibility, thereby modulating access to the active-site cavity. Similarly, the N96G substitution shortens the side chain in the αB–βC loop, possibly affecting intersubunit packing or allosteric communication between the small and large subunits (Fig. 4 and *SI Appendix*, Fig. S8). The terminal SSu residues (124 and 125) remain unresolved in the current structural model, indicating inherent flexibility within this region. Collectively, these findings reveal that even minimal sequence divergence among SSu isoforms can exert localized structural effects that propagate to the LSu core, underscoring the evolutionary fine-tuning of Rubisco stability and activity.

### Comparison with Reported Structures Reveals Substantial Conformational Flexibility.

**Fig. 5.**
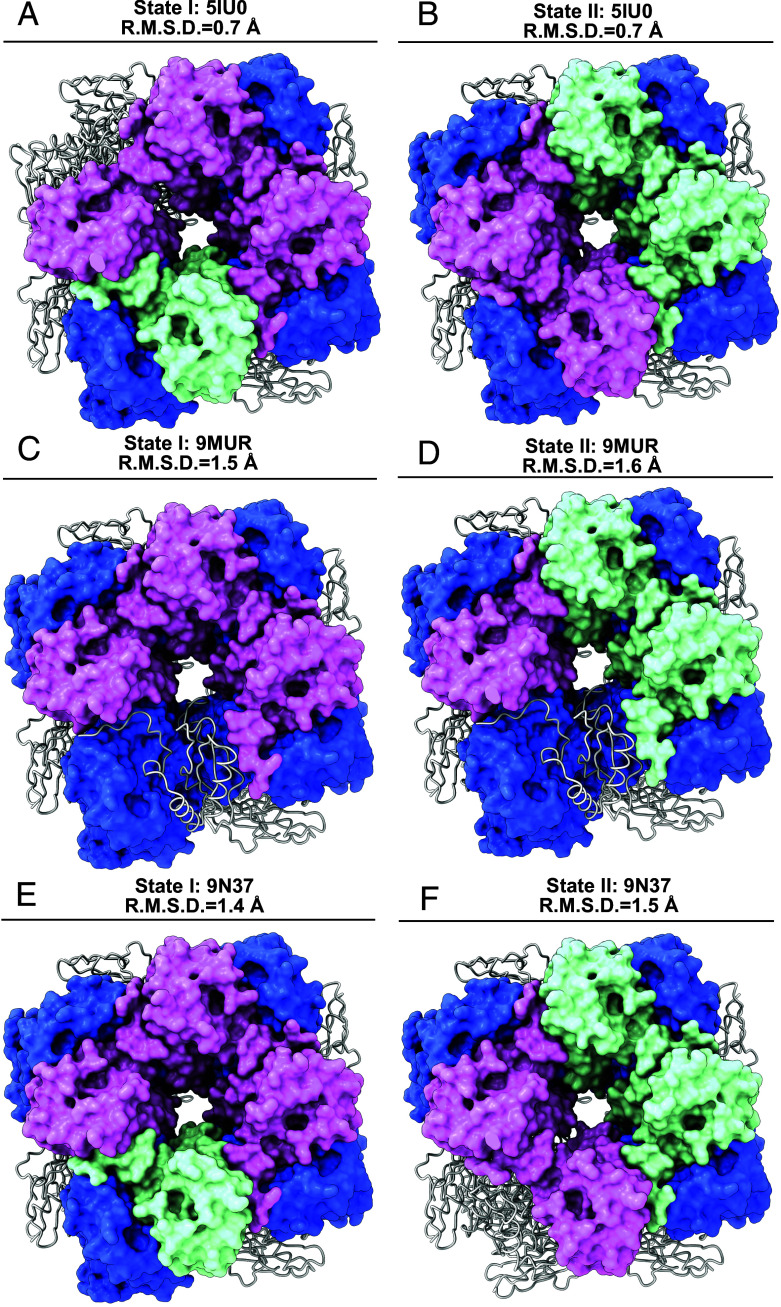
Structural comparison of State I and State II Rubisco with previously reported structures. Superimpositions of the cryo-EM structures of *A. thaliana* Rubisco State I and State II with available Rubisco models. In all panels, structurally identical regions are shown in a gray string. Variable regions are highlighted in color surface: SSu1A in green, SSu2B/3B in magenta, and LSu in blue. (*A* and *B*) Overlay of State I (*A*) and State II (*B*) with the *A. thaliana* Rubisco crystal structure (PDB ID: 5IU0, rmsd = 0.7 Å). (*C* and *D*) Overlay of State I (*C*) and State II (*D*) with the recently reported Rubisco structure 9MUR (rmsd = 1.5 Å and 1.6 Å, respectively). (*E* and *F*) Overlay of State I (*E*) and State II (*F*) with Rubisco structure 9N37 (rmsd = 1.4 Å and 1.5 Å, respectively).

To assess whether isoform-specific arrangements influence the global conformation of Rubisco, we performed pairwise superimpositions of State I and State II structures with three reference structures, 5IU0 (an *A. thaliana* crystal structure), 9MUR, and 9N37 (two structures reported in the accompanying paper, with 9MUR containing all eight SSu2B/3B and 9N37 containing all eight SSu1A) yielding rmsd values ranging from 0.7 to 1.6 Å (Fig. 5). When aligned on one face of the holoenzyme, the superimposed side shows good structural agreement (gray). In contrast, the opposite face exhibits greater positional shifts in both LSu and SSu regions. Local structural comparisons reveal discrete conformational differences in SSu1A (magenta) and SSu2B/3B (cyan/green) isoforms, as well as in specific large subunit domains (blue), with notable structural deviations. On the heterogeneous SSu side, both State I and State II adopt more extended conformations relative to 5IU0, yet are notably more compact than 9MUR and 9N37.

Importantly, States I and II represent the first Rubisco structures from *A. thaliana* with heterogeneous small-subunit composition, incorporating both SSu1A and SSu2B/3B isoforms. Notably, State II exhibits an unprecedented asymmetric distribution of SSu isoforms (2 SSu1A+2 SSu2B/3B on one face; 4 SSu2B/3B on the opposite face), a structural arrangement that has not been documented in any previous Rubisco structures. All prior *Arabidopsis* Rubisco structures, including 5IU0, contained homogeneous small-subunit populations, leading to speculation that heterogeneous populations cannot be accommodated in ordered crystals. Our cryo-EM structures definitively demonstrate that *Arabidopsis* Rubisco can assemble with mixed SSu isoforms in planta, resolving this long-standing question and establishing a unique architectural arrangement that distinguishes these states from all previously characterized Rubisco structures.

## Discussion

Our findings indicate that Rubisco in *A. thaliana* is not a uniform L_8_S_8_ complex but a mixed assembly where multiple SSu isoforms co-assemble in specific spatial arrangements. This challenges the longstanding assumption that each holoenzyme contains eight identical small subunits, instead revealing a structurally diverse complex. The most straightforward interpretation is that this compositional heterogeneity provides an additional layer of flexibility, allowing Rubisco to adapt its properties to different physiological or environmental conditions. For instance, *Arabidopsis* SSu “B” isoforms are upregulated at elevated temperatures (18), consistent with a role in enhancing stability and performance under heat stress.

While the majority of complexes detected by native MS/MS and resolved by cryo-EM exhibited mixed SSu composition, we cannot exclude the presence of a minor population of homogeneous L_8_S_8_ assemblies below the detection limit of our methods. In this context, the previously reported *A. thaliana* Rubisco crystal structure 5IU0, purified from a native source and interpreted as containing eight copies of a single low-abundance SSu isoform, likely represents one end of a continuum of possible SSu compositions under different physiological or experimental conditions. Our data suggest that heterogeneous assemblies predominate under the growth and purification conditions used here, but homogeneous complexes may also occur transiently or under alternative environmental regimes. Compositional heterogeneity of Rubisco appears pronounced in *Arabidopsis* and in several other species with diversified RbcS/SSu families (for example, the examples shown in *SI Appendix*, Fig. S2) but may be minimal in species where multiple *rbcS* genes encode identical mature SSus, for example, rice.

Recognizing that native Rubisco is built from a mixture of SSu isoforms may also help explain why some Rubisco-engineering efforts have produced confounding results. Many studies have reconstructed the enzyme with a single SSu variant or replaced plant small subunits with those from faster algal Rubiscos (6, 7). In most cases, these complexes do not perform as well as the native enzyme. Our findings suggest that these strategies might have missed a key point: In planta, Rubisco does not depend on a single “best” small subunit but on a carefully balanced ensemble. The unique kinetic effect of heterogeneity is demonstrated in our companion paper, which finds that compositionally heterogeneous Rubisco has an intermediate turnover rate but decreased substrate and inhibitor affinities relative to its pure counterparts (22). Therefore, engineering efforts must consider potential heterogeneity-related kinetic effects when introducing nonnative SSus.

The hypotheses arising from this work are now directly testable. Genome editing allows the deletion of specific *rbcS* genes, creation of defined isoform combinations, or swapping of promoters to modify their expression profiles. Examining the effects on Rubisco kinetics, assembly efficiency, and in planta photosynthetic performance would establish a direct link between structural heterogeneity and function. Complementary reconstitution in manageable hosts such as *Nicotiana* or *Chlamydomonas*, including existing hybrid Rubisco systems (23), could aid in distinguishing effects on biogenesis from those on catalysis and in determining the extent to which native-like mixtures are necessary to restore full activity.

Methodologically, the combined use of native top–down MS and cryo-EM provides a versatile approach for examining multi-isoform protein complexes in plants. Top–down MS identifies which proteoforms are present within intact assemblies, while cryo-EM shows how these variants are spatially organized in 3D. Together, these methods reveal structural heterogeneity that would otherwise be averaged out in bulk biochemical analyses or hidden during crystallization. The same framework should be applicable to other paralog-rich chloroplast machinery where isoform-specific functions remain poorly understood, including Rubisco activase, the Cpn60–RbcL chaperone system, and components of the TOC–TIC protein import pathway (4, 23–27).

More broadly, our findings suggest a conceptual shift. Enhancing enzyme function in plants may not be achieved by perfecting a single protein sequence, but by maintaining a group of closely related isoforms that collectively balance rigidity and flexibility. Such compositional plasticity would enable quick adjustment of enzymatic properties in response to changing environments, without the need for extensive rewiring of transcriptional programs or metabolic networks. From this perspective, Rubisco heterogeneity is not a defect to be eliminated but a feature that evolution has repeatedly favored and preserved.

## Materials and Methods

### Rubisco Sample Purification.

*A. thaliana* Col-0 chloroplast stroma was extracted by existing methods (19), and Rubisco complexes further enriched by concentrating with MWCO 100,000 kDa filter.

### MS.

#### Bottom–up MS.

Bottom-up proteomics analysis was performed using an Orbitrap Eclipse Tribrid Mass Spectrometer (Thermo Fisher Scientific). Data were acquired in a data-dependent acquisition mode employing a TopN method with HCD fragmentation and a 1h elution gradient HPLC method, as previously described (28). Raw MS data were processed and analyzed using MaxQuant (29). Peptide intensities were normalized across runs to account for technical variability.

#### Native MS.

Purified Rubisco was buffer exchanged into MS Buffer (200 mM ammonium acetate pH 8.0) using a centrifugal buffer exchange device (Micro Bio-Spin 6, Bio-Rad). Samples were introduced directly into the mass spectrometer using gold-coated capillary needles (prepared in-house). Data were collected on a Q-Exactive UHMR mass spectrometer (Thermo Fisher Scientific). The instrument parameters were as follows: capillary voltage 1.1 kV, S-lens RF 100%, quadrupole selection from 1,000 to 20,000 m/z range, collisional activation in the HCD cell 100 to 200 V, trapping gas pressure setting 7.5, temperature 200 °C, and resolution of the instrument 12,500. The noise level was set at 3 rather than the default value of 4.64. No in-source dissociation was applied. Data were analyzed using Xcalibur 4.2 (Thermo Scientific) and UniDec software packages.

#### Top–down MS.

The top–down analysis was also performed on the same instrument. The typical MS settings were spray voltage of 1.1 kV, source temperature of 200 °C. The SSu subunits were dissociated from the complex with 100 V using in-source fragmentation. The dissociated subunit peaks were selected using ion trap with a window of m/z 10. The selected ions were then further fragmented using HCD energy. The fragment ions were detected in the Orbitrap. The top–down mass spectra were manually validated in TDValidator (Proteinaceous) (30).

### Cryo-EM Sample Preparation.

For imaging Rubisco, a 2.5 µL of 5 mg/mL stroma sample was directly applied to glow-discharged holey carbon grids (Quantifoil Cu R1.2/1.3, 300 mesh), blotted for 18 s and then plunge-frozen in liquid ethane using a Vitrobot (Thermo Fisher). For imaging, all grids were then transferred into cartridges prior to data collection.

### Cryo-EM Data Collection.

The images were collected in superresolution mode at 81 K magnification on a Titan Krios equipped with a K3 direct electron detector (Gatan). The physical pixel size was 1.07 Å/pix (superresolution of 0.535 Å/pix). Each micrograph was exposed to a total dose of 37.8 e^−^/Å2 for 3.5 s and 40 frames were captured using SerialEM (31).

### Cryo-EM Data Processing.

The super-resolution image stack was aligned and binned by 2 using patch motion. The contrast transfer function (CTF) was estimated using patch CTF in cryoSPARC (32). A procedure for blob picker followed by 2D classification were applied to generate templates for automated template picking. Initially, 1,659,413 particles were selected after autopicking in cryoSPARC. Several iterative rounds of 2D classifications followed by ab initio and heterogeneous 3D classifications were performed to remove false picks and classes with unclear features, ice contamination, or carbon. The 3D classification analysis was then employed, resulting in two distinct classes of Rubisco images. Non-uniform refinement followed by local refinement with nonuniform sampling resulted in 2.87 Å and 2.97 Å resolution cryo-EM maps for two forms Rubisco based on the gold standard Fourier shell correlation (FSC 0.143).

### Model Building and Refinement.

Model buildings of Rubisco state I and state II were based on the cryo-EM maps, respectively. A predicted Rubisco structure using AlphaFold (33) was used and fitted into the corresponding density maps using Chimera (34). The subsequent model rebuilding was performed using Coot (35). Structural refinements were performed using the phenix.real_space_refine program (36) from the PHENIX suite (37). The final atomic model was evaluated using MolProbity (38). The statistics associated with data collection, 3D reconstruction, and model refinement are included in *SI Appendix*, Table S1.

## Supplementary Material

Appendix 01 (PDF)

## Data Availability

Cryo-EM maps and atomic coordinates data have been deposited in EMDB and PDB [EMD-70094 (39) and 9O4C (40) for state I and EMD-70090 (41) and 9O49 (42) for state II].
