# Supplementary material for: Small subunit isoform diversity underlies structural heterogeneity in native plant Rubisco
Source: Proc Natl Acad Sci U S A. 2026 Apr 15;123(16):e2519949123. doi: 10.1073/pnas.2519949123 (PMC13099656; doi:10.1073/pnas.2519949123)
Supplement: Supplementary file 1 — Appendix 01 (PDF) [file pnas.2519949123.sapp.pdf]

PNAS

Supporting Information for

Small subunit isoform diversity underlies structural heterogeneity in native plant Rubisco.

Thomas Reynolds[1#], Zhemin Zhang[2#], Dušan Živković[1,3], Steven Kelly[1], Jani R Bolla[1,3*]

[1]Department of Biology, University of Oxford, Oxford OX1 3RB, United Kingdom
[2]Department of Pharmacology, Case Western Reserve University, Cleveland, OH 44106, United States of America
[3]Department of Biochemistry, University of Oxford, Oxford OX1 3QU, United Kingdom

[#]These authors contributed equally
*Corresponding author: jani.bolla@bioch.ox.ac.uk

This PDF file includes:

Figures S1 to S8
Tables S1

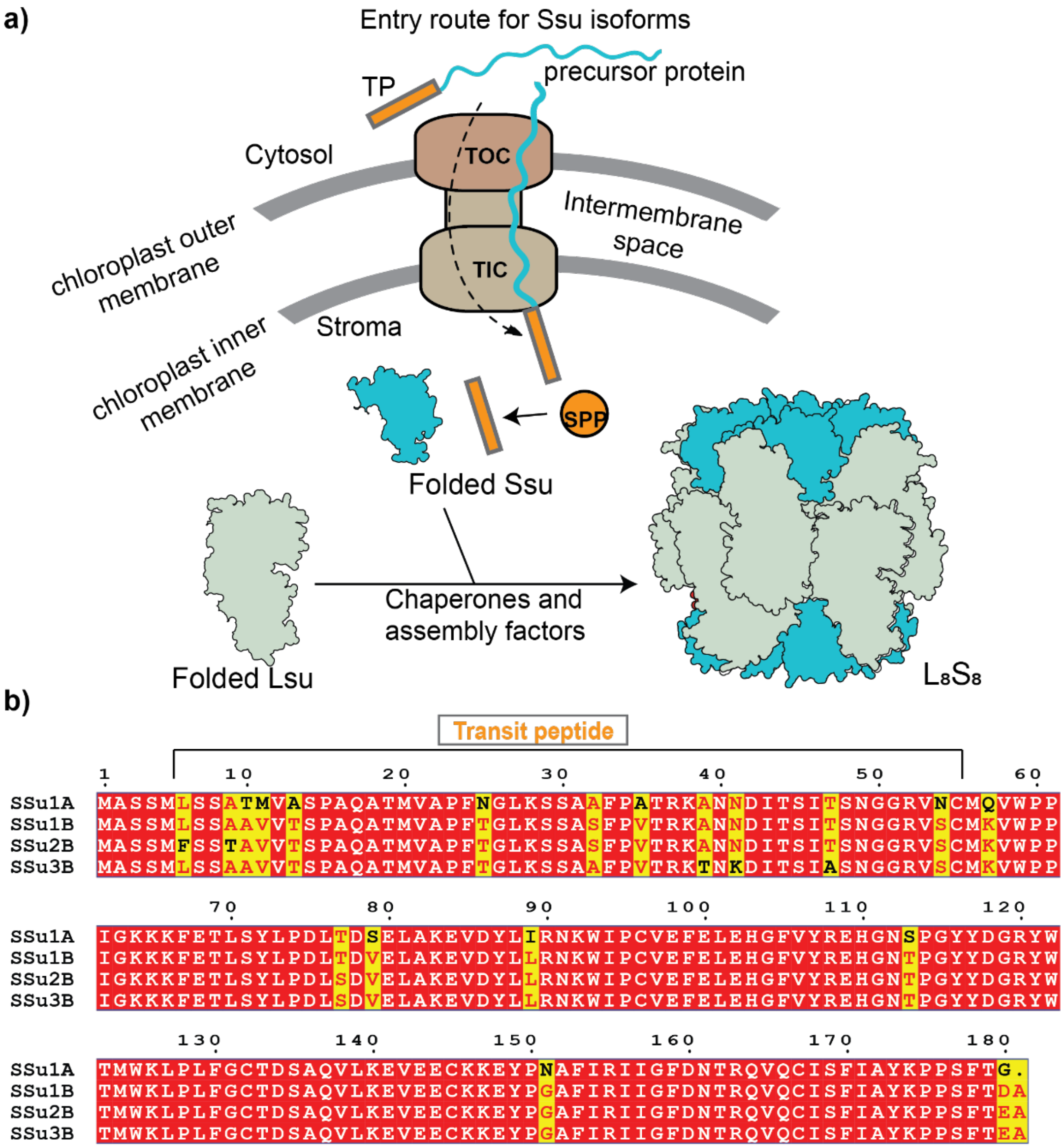

Figure S1. ***SSu isoforms are imported into chloroplasts to form functional Rubisco.*** a) Schematic illustrating the journey of the SSu from its site of synthesis in the cytosol to its assembly with the LSu in the chloroplast stroma to form the functional holoenzyme. SSu is synthesised in the cytosol as a preprotein with an N-terminal transit peptide (TP), which is recognised by the TOC–TIC protein import machinery and imported into chloroplasts. Once in the stroma, the TP is cleaved by the stromal processing peptidase (SPP), and the mature SSu is folded and assembled with the LSu into functional Rubisco with the assistance of several chaperones and assembly factors. b) Multiple sequence alignments of the different SSu isoforms in *A. thaliana*. Residues conserved

between isoforms are shaded in red, whereas positions that differ between isoforms are highlighted in yellow. Numbering corresponds to the full SSu sequences, including the transit peptide.

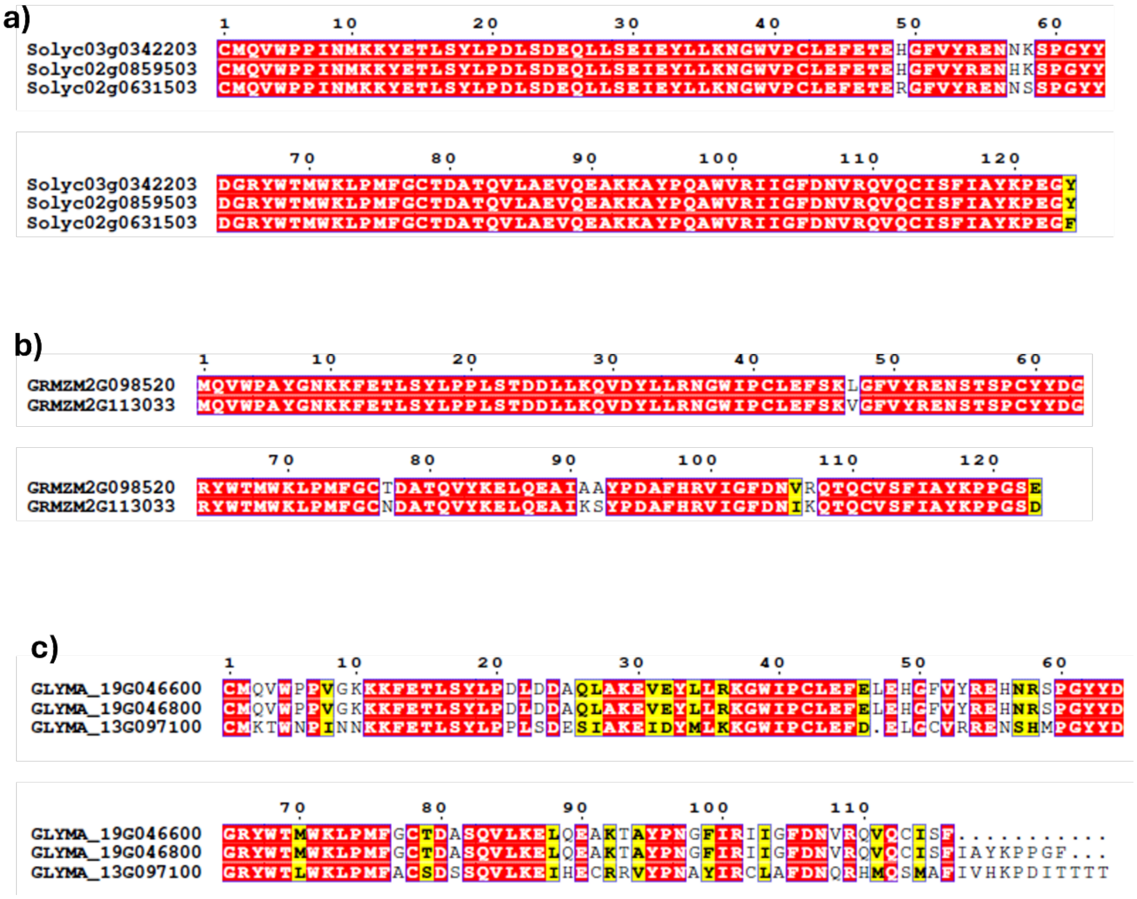

Figure S2: ***Conservation and diversification of Rubisco SSu isoforms in crop species***. Multiple sequence alignments of SSu isoforms from (a) tomato (Solanum lycopersicum), (b) maize (Zea mays), and (c) soybean (Glycine max). For each species, all annotated SSu isoforms are shown, note that transit peptides are not included. Residues conserved between isoforms are shaded in red, whereas positions that differ between isoforms are highlighted in yellow. Numbering corresponds to the mature SSu sequences. The alignments reveal that each species encodes closely related SSu isoforms that differ at a limited number of positions, analogous to the isoform-specific sites identified in *A. thaliana*.

Sequence	Protein Name	PEP*	Score**	Intensity
EHGNSPGYYDGR	RBS1A_ARATH	2.55E-183	167	6.04E+09
EYPNAFIR	RBS1A_ARATH	9.81E-14	114	8.93E+09
KEYPNAFIR	RBS1A_ARATH	5.57E-03	46	8.09E+07
KFETLSYLPDLTDSELAKEVDYLIR	RBS1A_ARATH	2.85E-21	52	1.22E+08
FETLSYLPDLTDSELAK	RBS1A_ARATH	3.67E-34	83	5.95E+09
KFETLSYLPDLTDSELAK	RBS1A_ARATH	3.52E-07	26	1.13E+07
KKFETLSYLPDLTDSELAK	RBS1A_ARATH	1.67E-19	91	7.07E+09
FETLSYLPDLTDVELAK	RBS1B_ARATH	2.50E-10	51	1.36E+09
KFETLSYLPDLTDVELAK	RBS1B_ARATH	1.31E-43	156	2.80E+09
KKFETLSYLPDLTDVELAK	RBS1B_ARATH	7.14E-23	100	4.37E+08
FETLSYLPDLSDVELAK	RBS3B_ARATH; RBS2B_ARATH	2.70E-22	95	4.98E+09
KFETLSYLPDLSDVELAK	RBS3B_ARATH; RBS2B_ARATH	2.03E-34	147	9.69E+09
KKFETLSYLPDLSDVELAK	RBS3B_ARATH; RBS2B_ARATH	6.17E-28	141	4.18E+09

Figure S3: ***Isoform-specific peptides identified by bottom-up proteomics***. Table listing the peptide sequences, corresponding protein (SSu isoform; UniProt names), posterior error probability (PEP), identification score, and MS signal intensity for *A. thaliana* SSu isoforms. Peptides highlighted in blue were used for relative quantification (three peptides per protein). PEP (posterior error probability) reflects identification confidence, with lower values indicating higher confidence; Score reports peptide–spectrum match quality, with higher values indicating better matches.

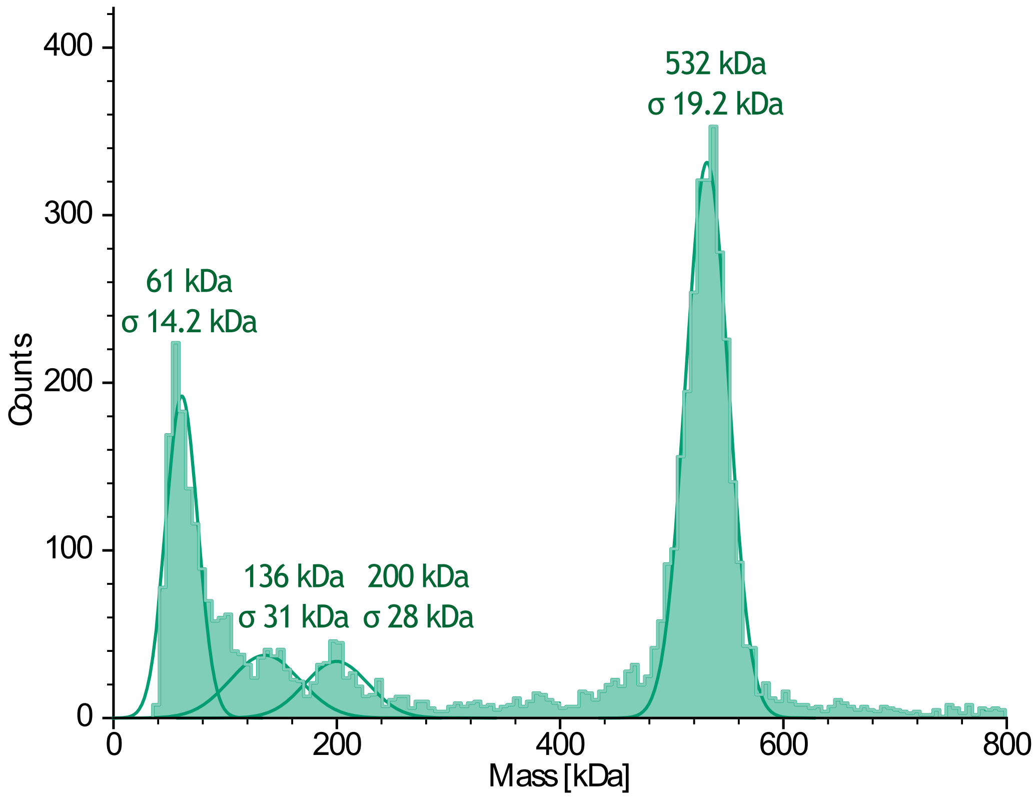

Figure S4: ***Mass photometry analysis of native A. thaliana Rubisco***. Mass photometry histogram of the native Rubisco preparation, with a Gaussian fit to the detected mass distribution (mean ± σ). The major peak at 532 ± 19.2 kDa corresponds to the native L_8S_8 Rubisco holoenzyme, whereas the minor lower-mass peaks likely represent co-purified proteins or protein complexes.

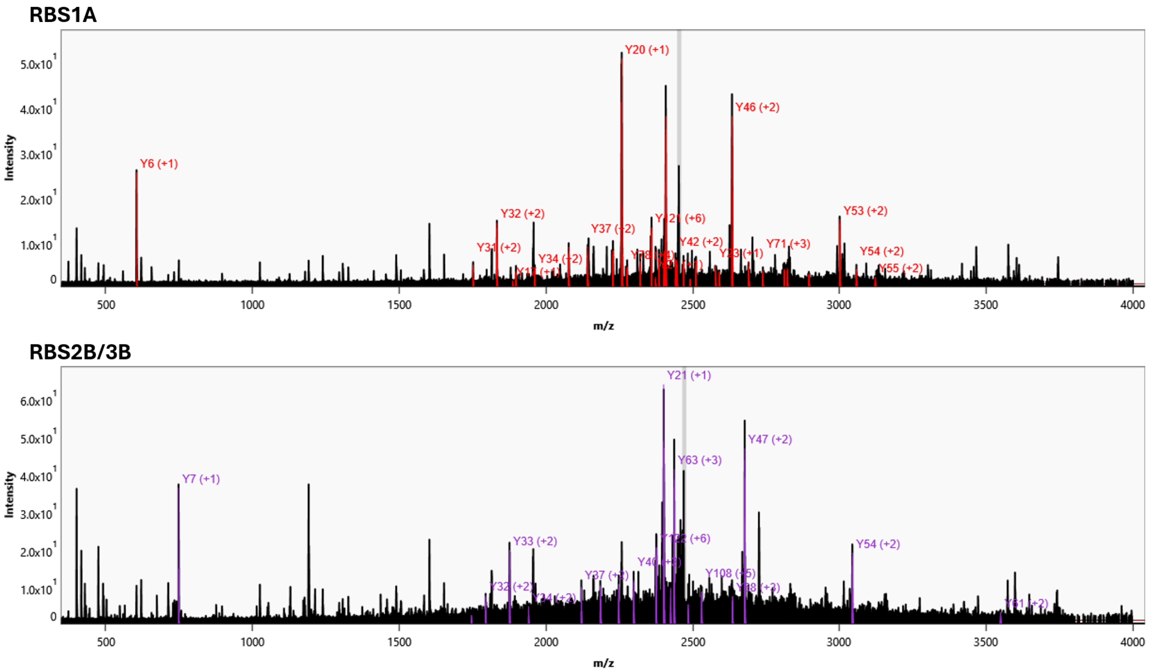

Figure S5: ***Native top-down fragmentation spectra of SSu isoforms***. Native top-down MS/MS spectra and peak assignments used for isoform identification. Top panel: y-type fragment ions specific to the SSu1A isoform are highlighted in red. Bottom panel: y-type fragment ions specific to the SSu2B/3B isoforms are highlighted in purple. These isoform-diagnostic fragments confirm the presence of both SSu1A and SSu2B/3B in the native Rubisco complex.

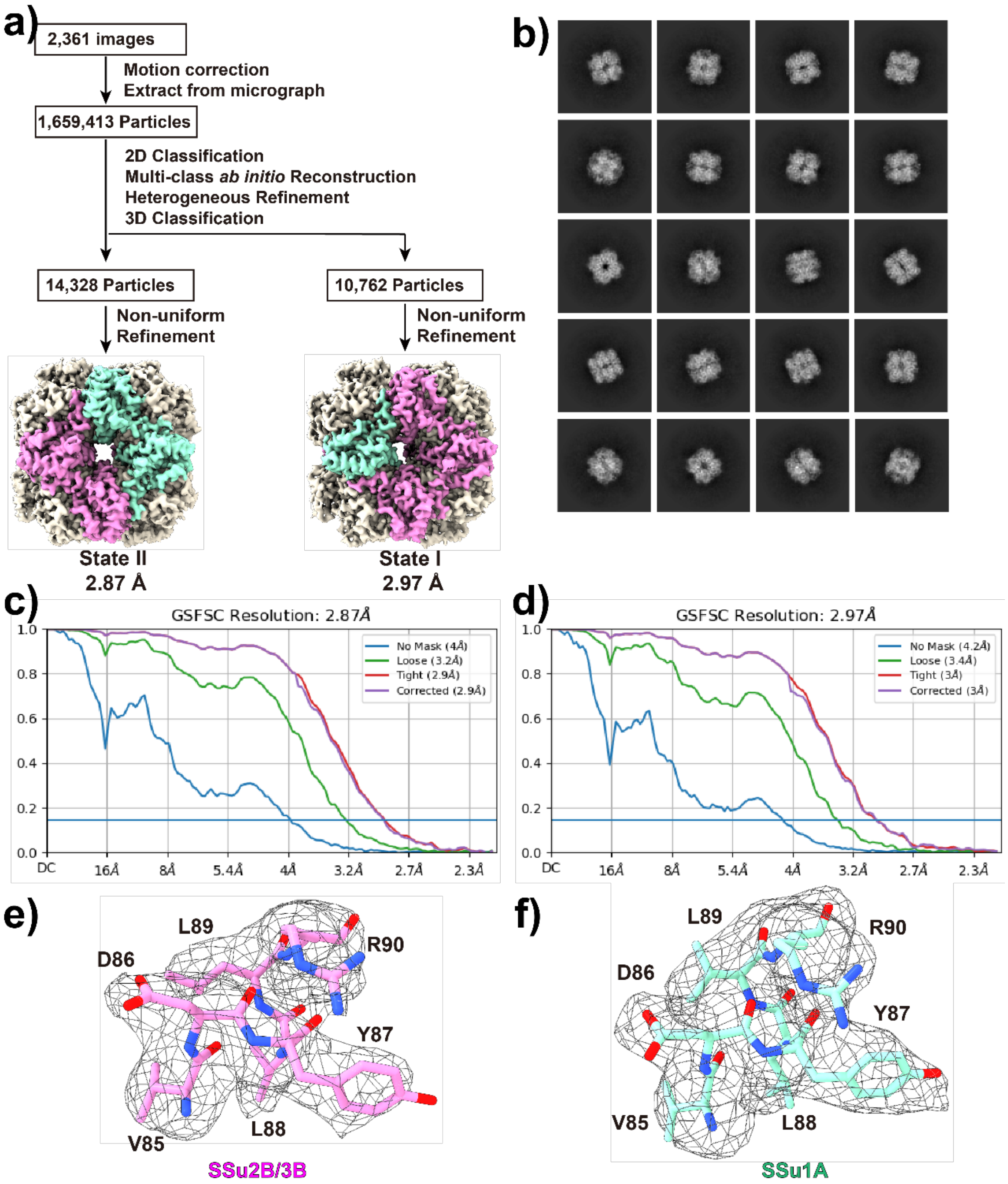

Figure S6: *Cryo-EM data collection, processing, and map quality for native Rubisco.* (a) Overview of the image-processing workflow, from motion-corrected micrographs to 3D classification and non-uniform refinement, yielding two final 3D reconstructions (State I and II) with the indicated resolutions. b) Representative 2D class averages showing particle views in multiple orientations. (c, d) Gold-standard Fourier shell correlation (FSC) curves for the State I (c) and State II (d) maps, with estimated resolutions indicated. (e, f) Local map density fitted with the corresponding atomic models for the SSu2B/3B isoform (e) and the SSu1A isoform (f), illustrating the quality of side-chain resolution.

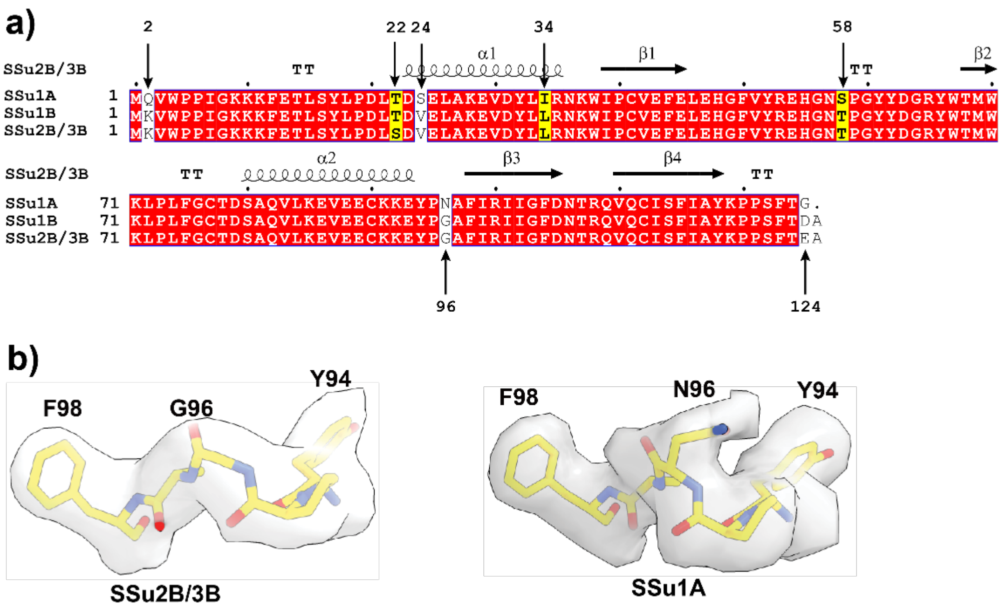

Figure S7: ***Identification of SSu isoforms in the cryo-EM maps***. a) multiple-sequence alignment of the mature forms of the *A. thaliana* SSu isoforms, with conserved residues shown in red and isoform-specific positions highlighted in yellow. b) representative cryo-EM density fitted with the corresponding atomic models: (left) density for the SSu2B/3B isoform, illustrating the presence of Gly96 alongside Phe98, and (right) density for the SSu1A isoform, showing Asn96 and Tyr94. The clear side-chain features at these diagnostic positions enable unambiguous assignment of the different SSu isoforms within the Rubisco complex.

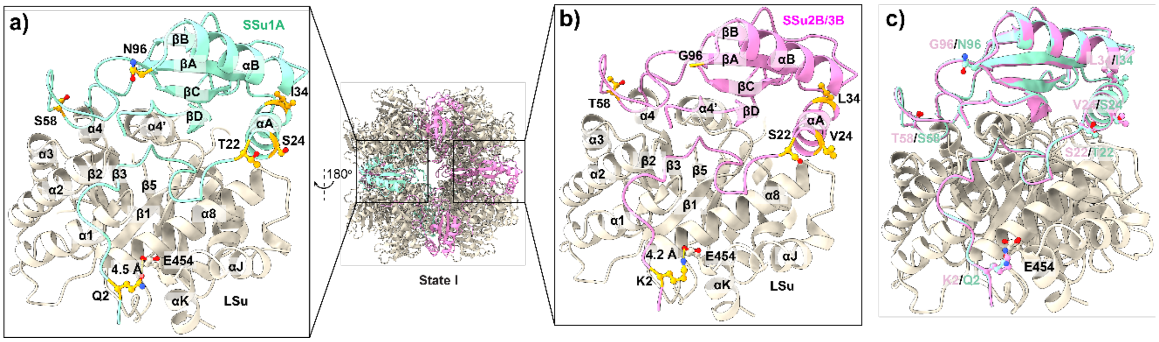

Figure S8: ***Isoform-specific interactions at the Rubisco LSu–SSu interface in State I***. a) Zoomed-in view of the interface between the large subunit (LSu, beige) and SSu1A (teal) in State I, showing contacts formed by the SSu N-terminus and β-barrel with helices and loops on the LSu. Selected interacting residues are shown as sticks, with the distance between the conserved K2–E454 ion pair indicated. b) Corresponding view of the LSu–SSu2B/3B interface (LSu, beige; SSu2B/3B, magenta), highlighting alternative side-chain identities and local rearrangements relative to SSu1A at analogous positions. c) Overlay of the LSu–SSu1A (teal) and LSu–SSu2B/3B (magenta) complexes, illustrating that while the LSu backbone is conserved, the two SSu isoforms adopt subtly different orientations and interaction networks at the interface.

Table S1. Rubisco cryo-EM data collection and refinement statistics.

**Data collection**		
Magnification	81,000	
Voltage (kV)	300	
Electron Microscope	Krios-GIF-K3	
Defocus (um)	-0.8 to -1.5	
Energy filter width (eV)	20	
Pixel size (Å)	1.07 (0.535)	
Total dose (e^-/ $Å^2$)	37.8	
Number of frames	40	
Number of micrographs	2,361	
Number of Initial particles	1,659,413	
**Refinement**	**State I**	**State II**
Number of total particles	10,762	14,328
GS-FSC Resolution (0.143, Å)[a]	2.97	2.87
Model composition		
Chains	16	16
Protein residues	4,418	4,424
Ligand	0	0
r.m.s.d.		
Bond lengths (Å)	0.004	0.003
Bond angles (°)	0.513	0.537
**Validation**		
MolProbity score	1.74	1.75
Clash score	9.03	7.82
Ramachandran plot		
Favored (%)	97.66	96.94
Allowed (%)	2.34	3.06
Disallowed (%)	0.00	0.00
CC Mask	0.80	0.82
